# Maxillary canine position of patients with non-syndromic craniofacial disorder: a retrospective evaluation of panoramic radiographs

**DOI:** 10.1186/s13005-023-00390-1

**Published:** 2023-10-09

**Authors:** C. Weismann, M. Lehmann, M. Aretxabaleta, B. Koos, M. C. Schulz

**Affiliations:** 1 Department of Orthodontics, University Hospital Tübingen, Osianderstr, 2-8, 72076 Tübingen, Germany; 2 Department of Oral and Maxillofacial Surgery, University Hospital Tübingen, Osianderstr, 2-8, 72076 Tübingen, Germany

**Keywords:** Unilateral cleft lip and palate, Bilateral cleft lip and palate, Tooth agenesis, Canine position, Robin sequence, Cleft palate, Orthopantomogram

## Abstract

**Background:**

The study evaluates the position and displacement tendency of unerupted maxillary canines in orthodontic patients with non-syndromic craniofacial disorders (CD) compared to a control (C) group.

**Methods:**

Canine position and displacement tendency were evaluated using panoramic radiographs (PAN) examined with parameters such as sector classification (sectors 1–5) and inclination angles (α and β). The displacement tendency was defined as the positioning of the tip in sectors 1 or 2, as well as its combination with increased angles (α > 30° and β > 39°). In addition, the correlation of the tooth position and agenesis, cleft side, and sex was assessed.

**Results:**

A total of 116 pre-treatment PAN, divided into the CD group (*n* = 50; mean age 8.32 ± 2.27 years) and the C group (*n* = 66; mean age 10.80 ± 2.82 years), were evaluated in this study. The sector classification showed no displacement tendency in both groups. Inclination angles α/β showed a statistically significant higher displacement tendency (*p* = 0.01) of the CD group (*n* = 5) on the right side, compared to healthy subjects (*n* = 1). Male CD patients had a statistically significant higher displacement tendency on the right side (*p* = 0.03). A statistically significant correlation between cleft and non-cleft-side (*p* = 0.03) was found.

**Conclusion:**

Patients with CD showed a statistically significant higher displacement tendency of the maxillary canine affected by the cleft side. The inclination angle was found to be the better predictor compared to the sector classification which should be considered in the orthodontic treatment planning.

## Background

Cleft lip and/or palate (CL/P) is one of the most frequent hereditary craniofacial disorders (CD), with a prevalence of 1:600 life-births [1]. The etiology of cleft malformations is multifactorial, possibly including both endogenous and exogenous factors [2], with a variating incidence according to the geographic location, socioeconomic status, sex, and ethnicity [3]. Different cleft formation combinations and severities can be found which can be also associated with other syndromes, e. g. Robin sequence (RS) (prevalence 11.3/100 000) [4]. RS involves the triad of mandibular retrognathia, glossoptosis, both symptoms resulting in the third, upper airway obstructions [5]. Additionally, RS is associated with a cleft palate (CP) in 80–90% of the cases showing the palate particularly narrow and high [6, 7]. The occurrence of a cleft is related with restrictions in oral hygiene, dental arch form deformation, oro-nasal fistulas, distinctive skeletal discrepancies between the jaws and dental anomalies [8–10].

It is estimated that the permanent maxillary canine is displaced and impacted in 1–2% of the population [11, 12], being the most frequently impacted tooth [13]. Furthermore, in 48% of the cases these are associated with other dental anomalies, e. g. microdontia, supernumerary teeth, enamel hypoplasia and transposition of teeth [14, 15]. Specifically in patients with CD, the prevalence of ectopic and impacted canines is ten to twenty times higher compared to patients without any CD [16–21]. Therefore, early diagnosis is particularly important as it can significantly reduce complications, e. g. root resorption of the lateral incisor in up to 12% of the cases, ankylosis and/or the necessity to surgically remove the affected tooth [22–24]. Moreover, early diagnosis would allow an early orthodontic treatment ensuring the proper alignment and adjustment of the dental arch [25].

Screening for canine impaction and the associated dystopia is usually performed between the age of 8 -10 years. This usually includes the palpation of the area, symmetry comparison with the opposite quadrant, evaluation of the tipping of the lateral incisor, and, if indicated, a radiographic evaluation [26]. The introduction of cone beam computed tomography (CBCT) in dentistry has contributed to a more exact localization of ectopic teeth and led to a more exact treatment planning [27, 28]. However, due to the lower radiation exposure the panoramic radiograph (PAN) remains the standard method of radiographic diagnostic. There are identifiable radiographic signs in PAN that allows to predict the position of the unerupted maxillary canines. Ericson and Kurol defined the examination criteria of ectopically erupting maxillary canines for PAN in 1987 [23, 24, 29, 30]. They described the mesio-distal location of the crown by two predictors of displacement, being the sector and the angulation of the tooth. For diagnostic purposes, it is desirable to have a reliable predicting parameter in PAN, for appropriate timing of the orthodontic intervention in order to decrease the need of surgical intervention, reducing complications, improving the tooth’s long-term prognosis and simplifying the orthodontic therapy [22, 23, 31–34]. Canine eruption, as well as their displacement tendency and according to predicting factors for healthy patients, has been extensively evaluated and discussed in the literature. In the opposite, literature addressing patients with CD is scarce and has not been sufficiently analyzed.

Therefore, this study aims to evaluate the position and displacement tendency of the unerupted maxillary canine in orthodontic patients with non-syndromic CD compared to healthy controls using predictors like sector classification of the crown position and angulation of the canine in pre-treatment PAN. In addition, the association of the tooth position between tooth agenesis, cleft side, and sex were determined. Thus, a null hypothesis was defined, stating that patients with CD have an increased displacement tendency compared to patients without congenital disorder.

## Methods

### Study design

This cross-sectional study was performed in the Department of the University Hospital Tübingen, Germany. It was approved by the institutional ethics committee of the University Hospital Tübingen (approval number: 498/2019BO2) according to the current version of §45 (3) 4 and §46/2a of the Federal State Hospital Act of Baden Württemberg: “The use of patient data of the hospitals own patients does not require the informed consent of the patients or their legal guardians” [35].

### Participants

The PAN data were obtained from the clinical records of the Department of Orthodontics at the University Hospital Tübingen. The sample size for the group of patients with CD was dependent to the number of patient cases in the Department during the time of the study. The timeline of recruitment was between 04/2019 and 10/2019. PANs considered for the study, were those taken at the initial time presentation to the department and those considered as part of the pre-treatment diagnostic.

The following inclusion criteria were applied:

- Age between 4 and 18 years. Patients younger than 4 years were excluded, considering that tooth development does not allow to identify the maxillary canine tooth position [36, 37], as well as that patients with a CD show a significant delay of tooth mineralization [38]. As tooth development and facial growth should be completed at the age of 18 years [39], patients older than 18 years were excluded.
- Non-syndromic CD: The diagnosis was confirmed by the neonatal medical picture or pre-operative record.
- Patients with a PAN according to the ALARA (as low as reasonably achievable) principles with an indication justifying radiation exposure within the course of orthodontic treatment.

The exclusion criteria were defined as follows:

- Additional associated complex CD (syndromes) or mental retardation, as some syndromes are associated with dislocation, impaction and malposition of (maxillary) canines.
- Patients younger than 4 years and older than 18 years.
- Radiographs with insufficient quality for diagnostic purposes, e. g. overexposure.

The collected participants were divided into two groups: One including patients with CD (CD group) and the control group (C group) consisting of patients without CD. Given that the CD group represented the presence of a non-syndromic CD, e. g. at least the cleft of the soft palate, it was composed of patients with CL/P and RS.

### Instruments for assessment

Sector classification and angulation angles α/β were used to examine the unerupted canines’ position and angulation in PAN. These two methods were applied as a predictor, as well as a tool to increase the ability to estimate a potential displacement.

### Sector classification of the maxillary canine

**Fig. 1:**
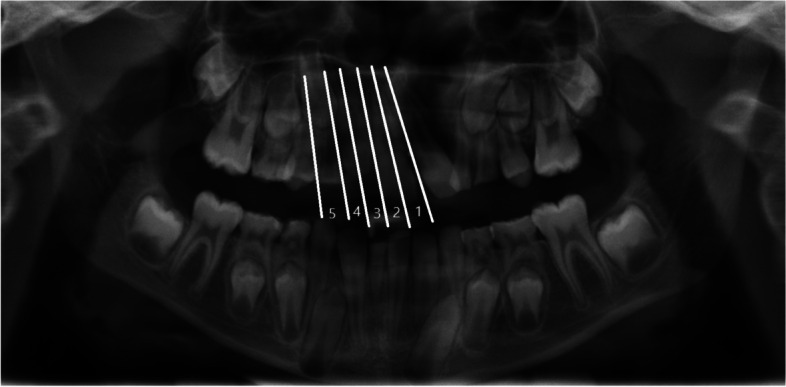
Exemplary panoramic radiograph with a schematic illustration of the right maxillary canine Sector (lines 1 to 5). The radiograph corresponds to a 10-year-old patient with an unilateral, left-sided cleft lip and palate, numeric hypoplasia of teeth 22 and 42, as well as a persistence of deciduous tooth 82

To assess the position of the maxillary canine, the projection of the canines’ cusp was divided into five zones, according to the sector classification inaugurated by Ericson and Kurol [30], shown in the schematic illustration of the PAN in Fig. 1. Sector 1 was oriented to the midline of the maxilla mesial to the central incisor, whereas sector 2 was defined by the longitudinal axis of the central incisor. In addition, sector 3 formed the distal root surface of the central incisor, sector 4 was defined as the distal root surface of the lateral incisor, and sector 5 was oriented towards the mesiodistal position of the future place of each tooth in the tooth row. Thus, an indication of the dislocation tendency of the canine was given when the tip of the canine was located in sectors 1 and/or 2.

### Inclination angle of the maxillary canine

**Fig. 2:**
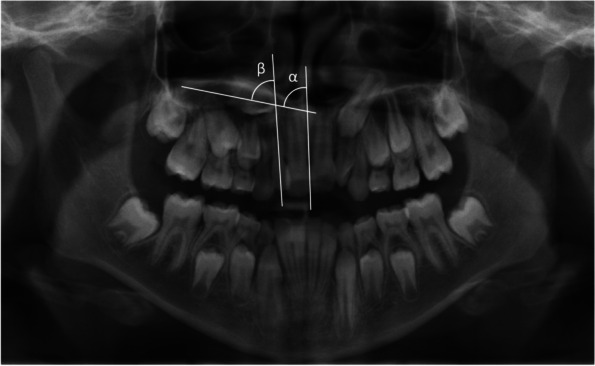
Exemplary panoramic radiograph with a schematic illustration of the inclination of the right, unerupted maxillary canine to the midline (angle α) and the longitudinal axis of the lateral incisor (angle β). Radiograph corresponding to a 11-year-old patient with RS and a cleft of the palate

The inclination angle of the maxillary canine in the frontal plane was assessed from angles α and β shown in Fig. 2 [30]. The latter was measured by the vertical inclination of the canines’ eruption path to the midline of the maxilla in between the central incisors (angle α) and to the distal tooth axis of the lateral incisor (angle ß). Thus, an indication of dislocation tendency of the maxillary canine is given when angle α is exceeding 30° in combination with angle β measures more than 39°.

### Interrater reliability

To assess the interrater reliability 37 randomly selected PAN, out of the total of 116 radiographs were evaluated by two examiners (ML, CW) applying the sector classification and the inclination angles α and β. The examination of the radiographs was independently performed by the examiners, both spatially and temporally separated. The examiners were experienced orthodontists of the Department of Orthodontics at the University Hospital Tübingen.

### Statistical analyses

The patient data, such as clinical records, were obtained from the electronic database. Subsequently, corresponding data was pseudonymized and processed in an Excel sheet (Microsoft Inc., Redmond, Washington, USA). The sample size was calculated with a two-sided two-sample t-test method by the Institute for Clinical Epidemiology and Applied Biometry at the University Hospital Tübingen. All statistical evaluations, descriptive statistics, and analysis were performed using JMP statistical software (Version 15.2.0, SAS Institute Inc., Cary, USA). Reliability of the interrater measurements was assessed by Cohen’s kappa. The distributions of the maxillary canine inclination and sector classification were studied by applying Pearson’s analysis. Normal distribution was evaluated by the Shapiro–Wilk test. The differences in the mean values between and in the two groups were evaluated using Student’s t-test for normally distributed data. In addition, other factors including tooth agenesis, sex and cleft side were statistically analyzed. For association tests of the sector classification and inclination angles α/β contingency analysis were used. The level of statistical significance was set up at an α of 5% (*p* = 0.05).

## Results

### Characteristics of study participants

The descriptive participant sample evaluation is shown in Table 1. From the initial screening of 120 participants, a total of 116 (50% male, 50% female) fulfilled the inclusion criteria, divided into the CD group (*n* = 50) and C group (*n* = 66). The PAN of four patients could not be used due to insufficient quality caused by motion artifacts and complex congenital CD, e. g. Apert syndrome. The mean age of all 116 patients was 9.73 ± 2.87 years. Participants from the CD group were statistically significant younger compared to C group (*p* < 0.05).

**Table 1:** Descriptive participant sample evaluation (n and percentage [%]) for both CD (craniofacial disorder) and C (control) groups. Means and their standard deviation (SD) are given for the age of the groups, whereas the participant number and percentage are given for sex, CD, and cleft formation distribution across the groups

*n* = 116	CD group (*n* = 50)		C group (*n* = 66)	
	*Mean*	*SD*	*Mean*	*SD*
**Age (years)**	8	2	11	3
**Sex**	*n*	*%*	*n*	*%*
Male	31	62	27	41
Female	19	38	39	59
**CD**	*n*	*%*	*n*	*%*
RS	5	10	/	/
CL/P	45	90	/	/
**Cleft location**	*n*	*%*	*n*	*%*
Unilateral	31	62	/	/
- *Left*	25	50	/	/
- *Right*	6	12	/	/
Bilateral	9	18	/	/
CP	5	10	/	/

*Abbreviations: SD,* Standard deviation*, CL/P* Cleft lip and/or palate*, RS* Robin sequence, *CP* Cleft palate only

### Interrater reliability

A very good degree of agreement between both examiners was assessed in the canine inclination angle β of the right side (Cohen’s kappa = 1.0) (Table 2). The canine inclination angle β of the left side indicated a good degree of agreement (Cohen’s kappa = 0.65). The remaining radiographic factors had a moderate degree of agreement for both examiners.

**Table 2:** Results of the evaluation of the interrater reliability between the two examiners (ML, CW) according to Cohen’s kappa coefficient

	**Cohen’s kappa-values**	**Degree of agreement**
Canine inclination Q1, α	0.48	moderate
Canine inclination Q1, β	1.0	very good
Sector Q1	0.44	moderate
Canine inclination Q2, α	0.48	moderate
Canine inclination Q2, β	0.65	good
Sector Q2	0.52	moderate

*Abbreviations: Q1* First quadrant*,* right upper jaw *Q2* Second quadrant*,* left upper jaw

### Sector classification and inclination angles

The sector classification showed no displacement tendency in both patient groups (Table 3). The most frequent canine position of patients with CD was on the right side in sector 5 (*n* = 26) and on the left side in sector 4 (*n* = 26). Compared to patients without CD sector 5 was the most frequent result in canine position on both sides (n__right_ = 43; n__left_ = 43). Comparing both groups, there was a statistically significant correlation of the sector results only on the left side (*p* = 0.02).

**Table 3:** Sector classification and inclination angles of the canine in the first and second quadrants of the different groups: CD (craniofacial disorder) group and C (control) group. Both groups were further divided considering sex and the CD group was further classified regarding the disorder and the cleft location. Statistical analysis was performed comparing both groups using Student’s t-test (F-ratio, p-value)

	**CD group**										**C group**			**F-ratio**	**p-value**
	*Total*	*Disorder*		*Cleft location*				*Sex*		*Total*		*Sex*			
		**RS**	**CL/P**	**L**	**R**	**B**	**CP**	**M**	**F**			**M**	**F**		
*Count*	*50*	*5*	*45*	*25*	*6*	*9*	*5*	*31*	*19*	*66*		27	39		
*Sector Q1*														1.99	0.16
** 1**	**0**	0	0	0	0	0	0	0	0	**0**		0	0		
** 2**	**0**	0	0	0	0	0	0	0	0	**0**		0	0		
** 3**	**6**	0	6	4	0	0	2	4	2	**5**		1	4		
** 4**	**18**	1	17	11	0	4	2	10	8	**18**		8	10		
** 5**	**26**	4	22	10	6	5	1	17	9	**43**		18	25		
*Sector Q2*														3.46	0.07
** 1**	**0**	0	0	0	0	0	0	0	0	**0**		0	0		
** 2**	**0**	0	0	0	0	0	0	0	0	**0**		0	0		
** 3**	**3**	0	1	2	0	0	1	2	1	**5**		1	4		
** 4**	**26**	3	3	12	3	5	3	14	12	**18**		8	10		
** 5**	**21**	2	1	11	3	4	1	15	6	**43**		18	25		
*Inclination Q1*														6.10	0.01*
** α > 30°**	**8**	1	7	3	1	2	1	5	3	**2**		0	2		
** β > 39°**	**5**	0	5	2	0	2	1	4	1	**1**		0	1		
*Inclination Q2*														2.34	0.13
** α > 30°**	**10**	0	10	7	0	2	1	8	2	**5**		1	4		
** β > 39°**	**5**	0	5	2	0	2	1	3	1	**5**		1	4		

*Abbreviation*: *RS* Robin sequence, *CL/P* Cleft lip and/or palate, *L* Left unilateral cleft, *R* Right unilateral cleft, *B* Bilateral cleft, *CP* Cleft palate, *M* Male, *F* Female, *Q1* First quadrant, right side upper jaw, *Q2* Second quadrant, left side upper jaw

Statistically significant differences are denoted by “*” (*p* < 0.05)

Regarding the inclination angles (Table 3), the α angle on both sides was more frequently increased in patients with CD than in healthy subjects. Patients with CD showed increased angles and, therefore a statistically significant higher displacement tendency of the right maxillary canine (*p* = 0.01) compared to healthy subjects. Regarding the inclination angles given for sex distribution, male patients with CD having a statistically significant increased dislocation tendency on the right side compared to the C group (n__CD_ = 4; n__noCD_ = 0; *p* = 0.03).

Using Pearson’s analysis, the CD group showed a correlation between displacement tendency and tooth agenesis, even though this was not statistically significant. On the right side, two patients had a displacement tendency and tooth agenesis, whereas three patients had a displacement tendency without tooth agenesis. However, on the left side, three patients exhibited a displacement tendency combined with tooth agenesis, while two patients showed a displacement tendency without agenesis.

Additionally, patients with a unilateral CL/P comparing cleft and non-cleft-sides regarding the sector classification and angle inclination of the maxillary canine (Table 4) were assessed. There was no statistically significant correlation between the sector position of the cleft and non-cleft-sides. Most canines were located in sector 5 of the cleft side, and showed no displacement tendency. Regarding the inclination angle, there was a statistically significant correlation between both sides (*p* = 0.03), having an increased number of α angles > 30° on the cleft side (*n* = 8) compared to the non-cleft-side (*n* = 3). In total, four cases had a displacement tendency, in two patients on the cleft side and in two on the non-cleft-side.

**Table 4:** Results of patients with unilateral CL/P showing the comparison between cleft side and non-cleft-side regarding the sector classification and inclination angles of the maxillary canine

*n* = 31	**Sector classification**						**Inclination**			
	*1*	*2*	*3*	*4*	*5*	*p-value*	*α* > *30°*	*β* > *39°*	*Total*	*p-value*
**Cleft side**	0	0	2	12	17	0.86	8	2	2	0.03*
**None cleft side**	0	0	4	14	13		3	2	2	

Groups were compared by the Pearson’s analysis. Statistically significant differences were denoted by “*” (*p* < 0.05)

### Association tests of the used instruments

For association tests between the used radiographic methods, the patient groups were compared with each other by the sector classification separately on the left and right side and the inclination angle both sides were distinctly assessed. The results showed that there was a statistically significant correlation in between the inclination angles on both sides (right side *p* = 0.04, left side *p* = 0.01) and the sector classification on the left side (*p* = 0.02). However, for the right side, no statistically significant correlation was found (*p* = 0.35).

## Discussion

This study aims to evaluate the position and displacement tendency of the unerupted maxillary canines in orthodontic patients with non-syndromic CD compared to healthy controls, using predictors like sector classification of the crown position and angulation of the canine in the pre-treatment PAN. In addition, the association of the tooth position and tooth agenesis, cleft side, and sex were determined. Thus, a null hypothesis was defined, stating that patients with CD have an increased displacement tendency compared to patients without congenital disorder. This is a novel approach regarding the sample group of patients with CD, e.g. RS and CL/P in this study. Currently, there are no literature reports about canine position and displacement tendency in patients with CD.

Considering the sector classification, no displacement tendency for the maxillary canine was observed in both groups. Meanwhile, the inclination angle yielded a statistically significant higher displacement tendency in patients with CD compared to the healthy controls. Thus, the null hypothesis was fulfilled. Several studies have shown that the sector score parameter in combination with the angulation of the canine to the lateral incisor and the midline were powerful and reliable values [30, 40]. Thus, they recommend that the linear angle measurement in PAN should be combined with the additional assessment of the canine sector position [21, 41, 42]. Despite this, the results of the current study showed a higher correlation between the inclination angles than the sector classification. The inclination angle was proved to be a reliable predictor for the displacement tendency of the maxillary canines, certainly in the CD group given the presence of a CL/P. In this scenario, the authors recommend prioritizing the use of inclination angles as a predictor of the canines’ impaction and using the sector classification as a supplementary. The combination of both methods should increase the estimation of a tooth impaction at the start of orthodontic treatment [34].

In the current literature, the alpha angle according to Ericson and Kurol [30] is the most frequently used parameter to determine the angulation of the canine in patients with CL/P [17, 43]. Hereman et al. set the predictive cut-off value in patients with CL/P at an angle between the canine and the midline higher than 23.8 degrees [44]. The group of Russel and McLeod even increased this value with an angle of more than 45 degrees [45]. Rizell et al. used a modification of the angulation and sector measurements of Ericson and Kurol adapted to dental anomalies occurring in patients with CL/P [30, 46]. They have shown that cleft canines and especially the ones requiring surgical exposure had an increased angulation. These results are in line with this study, as increased alpha angles in patients with CD, particularly on the cleft side were observed. This could be the result of the angle building axis which is the midline of the maxilla in between the central incisors. As this midline is frequently affected by deviations caused by the cleft or tooth agenesis, it can influence the angulation of the line, resulting in an increased alpha angle. In contrast, the beta angle is not affected by a midline deviation, since it is defined by the canine position and the lateral incisor. Therefore, applying the alpha angle proved to be more accurate than the beta, certainly in patients with a CD. This finding goes in line, by regarding the difference in interrater reliability for alpha and beta angles. The beta angle had a superior degree of agreement compared to the alpha angle, meaning that it was easier for the examiner to mark on the radiograph or that it was less affected by the used method. In contrast to this, the alpha angle was more affected by the examiner and, therefore more sensitive considering the presence of anomalies like a cleft or angulation misalignment of the angles’ building involved tooth. Concerning the reliability of the kappa coefficient in the interrater agreement in radiology, it is described as a meaningful value, as it does not account for agreements due to chance alone [47].

Generally, in this study cleft-sided canines have shown a higher risk for dislocation and malposition compared to the non-cleft-sided ones. Not only this latter has to be considered in orthodontic treatment of patients with CD, also the correlation with tooth agenesis [48]. Several authors described that displacement and impaction coincide with tooth agenesis, specifically regarding the lateral incisor [21, 45, 49]. On the one hand, following the guidance theory, this tooth has a crucial role in guiding the maxillary canine to its correct position. On the other hand, the absence of the lateral incisor is frequently associated with the occurrence of a cleft, certainly on the cleft side. This tooth agenesis is more a characteristic sign of a CD and could consequently lead to a displacement of the maxillary canine.

In the current study, the location of the canines was assessed using visual diagnosis of the examiners without an exact standardized scaling of the PAN. In a previous study, tooth agenesis of patients with CL/P or RS were assessed by the same two examiners, and comparably to the current study, the kappa value showed a moderate degree of agreement [48]. This study was performed under the same conditions as the current one. The PANs were analyzed by both examiners independently, in separate rooms, and at different times. To assess the exact canine position, regardless of the reliability and bias of the examiner, three-dimensional images seem to be the more reliable and ideal radiographic technique. In patients with CD, three-dimensional images are performed routinely to evaluate the bone dimensions of the alveolus and palatal cleft. Indications for three-dimensional radiographic imaging are the assessment of bone volume before and after bone grafting or setting skeletal bone anchorage screws for the use of an orthodontic appliance [50]. In combination with these examinations, it is useful to assess the position and the potential agenesis of the teeth to avoid additional images, increased radiation exposure, as well as costs. This means that interdisciplinary therapy can be planned based on one three-dimensional image by providing benefits to the patient and therapists.

Considering the study design, certain limitations need to be mentioned. Regarding the age distribution of the compared patient groups, subjects with CD were statistically significant younger than the healthy ones. This fact could be a bias in the correlation between both groups. Unfortunately, as there is a higher tendency for an earlier orthodontic treatment for patients with CD, this limitation cannot be overcome [51]. The orthodontic treatment starts around the age of four to five years and therefore, the pre-treatment PAN is taken at a younger age compared to orthodontic patients without CD. However, the recommended therapy for patients without CD is set at the beginning of the first transitional stage, which is around the age of six to seven years. Aside from that, the literature describes that the displacement of a maxillary canine is regularly detected at an age of around ten years [52]. Before this age, it can be diagnosed by a missing lateral incisor, asymmetry in tooth eruption of the dental arch, conspicuous familial history, and, also while considering the above-mentioned information, also a radiographic diagnostic. This radiographic diagnosis can happen at a later time point during orthodontic treatment by an attentive orthodontist. Therefore, eliminating the mentioned bias is complex, as more importance was given to employ a pre-treatment PAN that exposed the position of a non-treated canine for the current study. After the therapy starts, e. g. extraction of the primary canine or reducing the crowding of the dental arch, approximately sixty to eighty percent of the canines with a displacement tendency erupt spontaneously [31, 53, 54]. This is a fact, which would have biased the results of the current study, when PANs during orthodontic treatment would have been taken for study purpose. Finally, the obtained sample size was limited but this was given the monocentric character of this study and the low prevalence of the studied patient population.

This is a cross-sectional retrospective study, based on data obtained from the clinical records evaluating the final canine displacement. In the future, the same canine displacement parameters should be evaluated in a prospective study design, both at the beginning of the observation period and towards the end, to evaluate the eruption position and the potential occurrence of a displacement.

## Conclusion

In conclusion, the results indicated that a higher displacement tendency of the unerupted maxillary canine occurred in patients with CD than in those without. This was statistically significant affected by the occurrence of a cleft and was found to be statistically significant more frequent in male patients with CD on the right side. The current study found the inclination angle to be a superior predictor for the displacement tendency of the maxillary canines, especially for the CD group given the presence of a CL/P. In this scenario, the authors recommend prioritizing the use of inclination angles as a predictor of canines’ impaction and using the sector classification as a supplementary. These findings should be considered in the orthodontic treatment planning of patients with CD, for a more rigorous monitoring of the canine position and eruption on the cleft side by radiographic diagnostic.
